# Update on the treatment of multisystem inflammatory syndrome in children associated with COVID-19

**DOI:** 10.2217/fvl-2022-0048

**Published:** 2023-01-19

**Authors:** Fangyuan Long, Shiheng Zhu, Zeguang Wang, Shungeng Zhang, Jinlong He, Xinbin Ge, Jun Ning

**Affiliations:** ^1^Department of Physiology, Jining Medical University, 133 Hehua Rd, Jining, 272067, China; ^2^Department of Paediatrics, Affiliated Hospital of Jining Medical University, Jining, Shandong, 272029, China

**Keywords:** cardiac dysfunction, COVID-19, gastrointestinal symptoms, hemadsorption, intravenous immune globulin, MIS-C, SARS-CoV-2

## Abstract

In late 2019, SARS-CoV-2 was detected in China and spread worldwide. In rare cases, children who were infected with COVID-19 may develop multisystem inflammatory syndrome (MIS-C), which could have higher mortality than COVID-19 itself. Therefore, diagnosis and management are critical for treatment. Specifically, most of the initial treatment options of MIS-C choose intravenous immunoglobulin (IVIG) and steroids as the first-line treatment for patients. Moreover, antagonists of some cytokines are used as potential future therapeutics. Of note, therapeutic plasmapheresis can be used as a treatment for refractory severe MIS-C. We believe that each patient, especially those with comorbid conditions, should have individualized treatment based on both multidisciplinary consensus approach and expert opinion.

The ongoing COVID-19 pandemic, caused by SARS-CoV-2 has had a profound impact on global health. As of 12 January 2022, the COVID-19 pandemic had affected nearly 310 million people and resulted in over 5.5 million deaths worldwide [1]. In its early stage, severe COVID-19 associated with mortality is found in adults, the elderly, and patients with comorbidities, such as cardiovascular disease, chronic lung disease and diabetes [2]. Children are considered to have mild symptoms because of their low pediatric hospitalization and low mortality [3]. The reasons to explain the low incidence in children are manifold. With the increase of age, the functions of natural killer cells, macrophages, neutrophils and T lymphocytes in the immune system of adults are impaired, and more severe clinical manifestations may occur [4–7]. Vaccination in children can induce specific epigenetic and metabolic modifications of cells that can generate a more effective immune response when pathogens invade [8,9]. Children have less exposure to outdoor environments and therefore lower exposure to pathogens [10]. This difference can also be explained by the expression levels of SARS-CoV-2 cellular receptors and co-receptors in children and adults. ACE2 is the main functional receptor for viruses to enter host cells. It is worth noting that the expression level of ACE2 in the lungs of children is lower than that of adults [11]. On the other hand, the levels of androgen and androgen receptors in children under 12 years of age are lower than in adolescents and adult men, which causes decreased TMPRSS2 levels regulated by them in children [12]. Thus, low expression of ACE2 and TMPRSS2 may play a potentially protective role in the severe COVID-19 infection in children.

With the increase in the number of infections, since April 2020, some children have been observed to have a fever, gastrointestinal symptoms, cardiac dysfunction, multiple organ failure and other characteristics [13]. Initially, the cause of the disease could not be determined, but soon these cases of Kawasaki-like syndrome and excessive inflammatory response were found to be associated with COVID-19. According to the guidelines of the CDC, this condition is named ‘multisystem inflammatory syndrome in children (MIS-C)’, which is identified by fever, rash and gastrointestinal symptoms following SARS-CoV-2 infection [14]. It is interesting that most of those children had tested negative but positive antibody levels were presented in the clinic [15–18]. Therefore, we recommend that MIS-C be considered a post-viral inflammatory disease rather than a COVID-19 complication.

Table 1 outlines the Royal College of Paediatrics and Child Health's (RCPCH) [19], WHO [20], and CDC's [21] case definitions of MIS-C. The criteria described in the RCPHC case definition are persistent fever (duration not defined), inflammation (neutrophilia, lymphopenia, elevated CRP, elevated IL-6 and IL-10 level, etc.) and evidence of single- or multi-organ involvement (such as cardiac, respiratory, renal, gastrointestinal or neurological), along with other clinical and laboratory findings (e.g., elevated troponin, abnormal fibrinogen levels and high D-dimers), an electrocardiogram and imaging results.

**Table 1.** The criteria and case definition of MIS-C by Royal College of Paediatrics and Child Health, Centers for Disease Control and Prevention and World Health Organization.

	Case definition		
RCPCH	Age	Child, age not defined	
	Clinical presentation	Fever	Persistent, duration not defined
		Laboratory evidence of inflammation	Neutrophilia
			Lymphopenia
			Low albumin
			Elevated CRP
			Elevated IL-6 and IL-10 level
			High D-dimer
			High ferritn
			Thrombocytopenia
		Multisystem organ involvement (≥2)	Shock
			Cardiac
			Respiratory
			Renal
			Gastrointestinal
			Neurological
WHO	Mucocutaneous manifestations	Classic or incomplete KD	
	Acute gastrointestinal problem	Diarrhea, vomiting or abdominal pain	
	No other obvious microbial cause	Included	
	Evidence of COVID-19	May be positive or negative	
	Age	0 to 19 years with fever for ≥3 days	
	Clinical presentation	Mucocutaneous inflammation signs or bilateral non-purulent conjunctivitis or rash	
		Hypotension or shock	
		Features of pericarditis, valvulitis, coronary abnormalities, or myocardial dysfunction	
		Coagulopathy	
		Acute gastrointestinal problems (abdominal pain, diarrhea, or vomiting)	
	Elevated inflammatory markers		
	No other microbial cause of inflammation, such as bacterial sepsis, streptococcal, or staphylococcal shock syndromes		
	COVID-19 infection (antigen test, serology, or RT-PCR), or the possibility of contact with COVID-19 patients		
CDC	Age	<21 years	
	Clinical presentation	Fever	≥24 h
		Laboratory evidence of inflammation	Elevated CRP
			Elevated ESR
			Elevated fibrinogen
			Elevated procalcitonin
			Elevated D-dimer
			Elevated ferritin
			Elevated LDH
			Elevated IL-6 level
			Neutrophilia
			Lymphocytopenia
			Hypoalbuminemia
		Multisystem organ involvement (≥2)	Cardiovascular
			Renal
			Respiratory
			Hematologic
			Gastrointestinal
			Dermatologic
			Neurologic
		Evidence of clinically severe illness requiring hospitalization	
	Recent or current COVID-19 infection (by serology, antigen test, or RT-PCR) or exposure (within four weeks before the onset of symptoms)		
	No alternative plausible diagnoses		

ESR: Erythrocyte sedimentation rate; KD: Kawasaki disease; LDH: Lactate dehydrogenase; RCPCH: Royal College of Paediatrics and Child Health; RT-PCR: Real-time polymerase chain reaction.

MIS-C and Kawasaki disease (KD) are similar in clinical presentation but do not cause the same changes *in vivo* and can be differentiated from each other in the following ways [22]. In terms of pathogenesis, MIS-C is positive for SARS-CoV-2 serology, while KD tests are negative. In terms of pathogenesis, MIS-C patients had high expression of CX3CR1 in Vβ21.3+ T cells with significant specificity of cell subpopulation expansion consistent with superantigen-mediated immune system activation, but there was no clear evidence of superantigen occurrence and expression in KD [23]. In terms of human multisystem involvement, MIS-C is often associated with hematologic abnormalities such as elevated acute phase reactants or even shock, and multi-organ involvement, such as prominent gastrointestinal symptoms with abnormal cardiac function. In terms of disease follow-up, there is evidence that MIS-C has enhanced autoimmune signaling and an increased likelihood of recurrence requiring long-term follow-up, whereas KD rarely recurs and is followed-up significantly less frequently than MIS-C [22].

MIS-C is a novel syndrome, and studies on it still vary widely. The purpose of this review is to compare and summarize the current treatment of MIS-C, focusing on the treatment of clinical manifestations, to provide a reference for the clinical diagnosis and treatment of MIS-C.

## Pathogenesis of MIS-C

**Figure 1.**
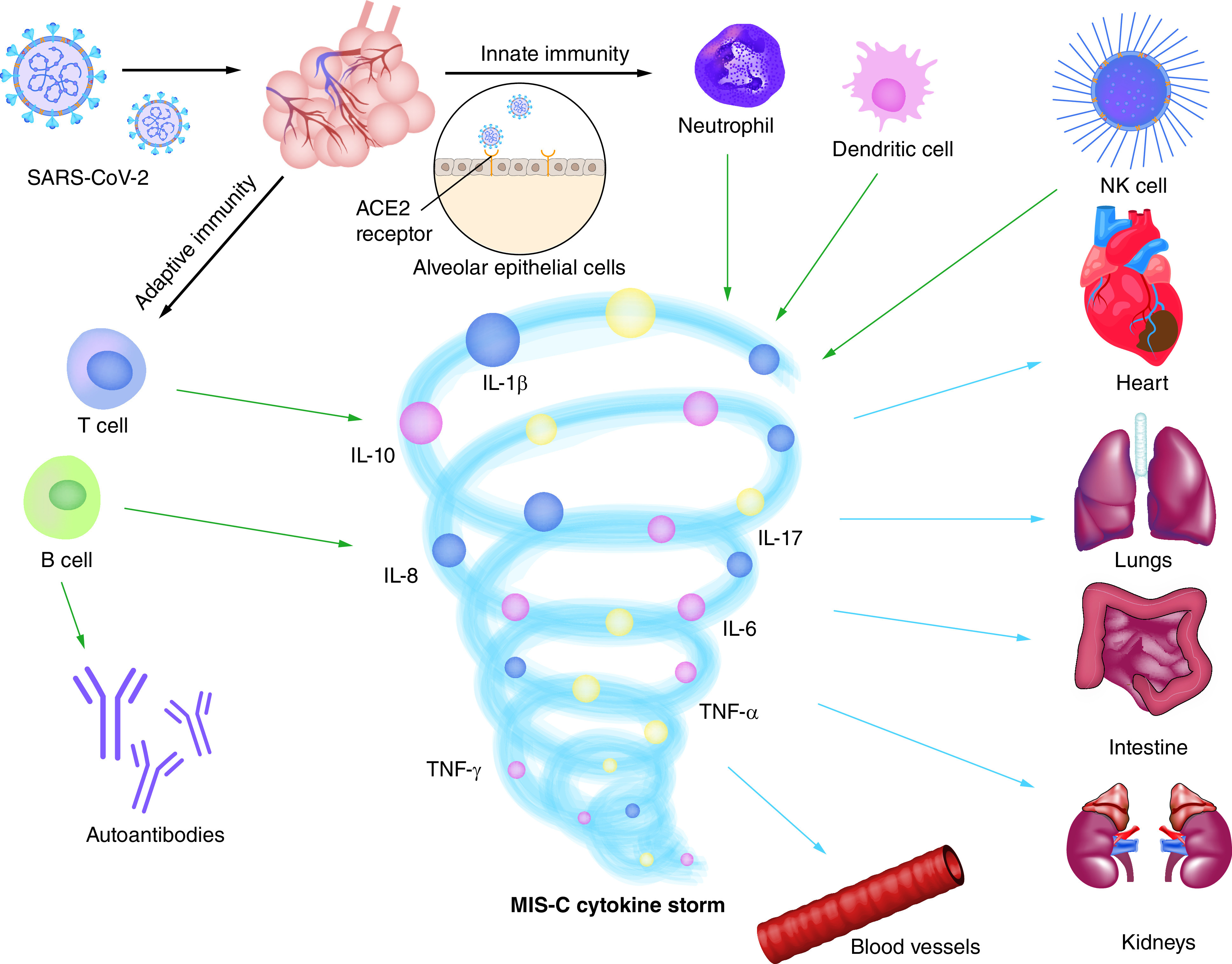
Potential pathogenesis of MIS-C. MIS-C: multisystem inflammatory syndrome; NK: Natural killer.

Although the MIS-C usually appears 2–4 weeks after infection with SARS-CoV-2 [24], the pathogenesis of MIS-C is not clear. It may be a delayed immune phenomenon associated with inflammation [25]. Normally, SARS-CoV-2 invades the body via nasopharyngeal cells, where the S protein of the virus interacts with host ACE2 receptors, and host cells and innate immune cells exert intrinsic immunity through pattern recognition receptors [26]. Moreover, MIS-C can be mediated by both humoral and cellular immunity [27]. And it shows that IgG antibodies and activation of CD8^+^ T cells present persistent elevation in critically ill children [28]. Germinal center-matured antibody secreting cells develop into long-lived plasma cells and memory B cells, providing higher and more persistent titers of virus-specific antibodies [29]. In addition, more naive T cells are present in young children, and, more importantly, the presence of lectin-like receptors on cytotoxic T cells maintains specific CD8^+^ T cells expressing high levels of cytotoxic mediators [26]. The SARS-CoV-2 infection triggers the release of large amounts of cytokines including interleukin-1 β (IL-1β), IL-6, IL-8, IL-10, IL-17, TNF-α and IFN-γ [30], which mediate a hyperinflammatory state which causes fever and systemic multisystem dysfunction in critically ill children (Figure 1) [27]. For example, the etiology of myocardial injury because of MIS-C may include acute viral myocarditis and ischemia due to coronary artery involvement [28]. Patients usually have prior cardiovascular disease, and after viral infection, small numbers of damaged cardiomyocytes can sustain immune activation and tissue inflammation, eventually leading to dilated cardiomyopathy and fibrosis [31]. In addition, cytokine storms as mentioned above can lead to coronary artery dilation in affected children [32]. MIS-C can also exhibit gastrointestinal symptoms, which may be attributed to the presence of numerous ACE2 receptors in the small intestine and colon [33]. The mechanism of acute kidney injury caused by MIS-C is thought to be tubular damage and podocytosis, inflammatory processes, hemodynamic instability, and vascular endothelial dysfunction due to an abnormal immune response to the virus [34]. The occurrence of MIS-C may be related to the genetic defects of children to a great extent. In a prospective gene sequence study, 3 of 18 patients (17%) were diagnosed with genetic defects. In addition to SOCS1 haploid defect, the children also had defects in X-linked apoptosis inhibitor (XIAP) and CYBB [35].

## Treatment of MIS-C

### Basic treatment of MIS-C

The treatment of MIS-C has continued to evolve during the pandemic. Some children who present with only fever, rash and systemic inflammation without other organ damage require only close observation in an outpatient setting [36]. Most patients with moderate and severe phenotypes are treated first with intravenous immunoglobulin (IVIG) and glucocorticoid (GC) therapy. Additional therapeutic interventions rely on the severity of the disease and the response to initial treatment. We discuss 6 studies with clinical data for patients with MIS-C and summarized clinical symptoms and treatment options of the patients (Table 2).

**Table 2.** Clinical manifestation and treatment of MIS-C.

Study	Case 1 [37]	Case 2 [38]	Case 3 [38]	Case 4 [38]	Case 5 [39]	Case 6 [40]	Case 7 [40]	Case 8 [41]	Case 9 [41]	Case 10 [42]	Aggregate
Sex	Female	Male	Female	Male	Male	Male	Male	Female	Female	Female	5/10 Male
Age (years)	7.7	6	6	12	17	14	13	3	10	11	Average 10.0 years
Ethnicity	NP	Asian	Afro–Caribbean	Afro–Caribbean	NP	NP	NP	Caribbean	NP	NP	–
RCPCH MIS-C Criteria^†^	+	+	+	+	+	+	+	+	+	+	10/10
Fever	Yes	Yes	Yes	Yes	Yes	Yes	Yes	Yes	Yes	Yes	10/10
Mucocutaneous	Yes	Yes	Yes	Yes	Yes	No	No	Yes	Yes	Yes	8/10
Organ-involvement	Yes	Yes	Yes	Yes	Yes	Yes	Yes	Yes	No	No	8/10
Gastrointestinal	Yes	No	Yes	Yes	Yes	Yes	Yes	Yes	Yes	Yes	9/10
Hypotension/shock	Yes	Yes	Yes	Yes	Yes	No	No	Yes	Yes	Yes	8/10
Abnormalities	Yes	Yes	Yes	Yes	Yes	No	No	Yes	No	No	6/10
Lab parameters	NP	D-dimer, albumin, Ferritin, CRP	D-dimer, albumin, CRP	D-dimer, albumin, Ferritin, CRP	Ferritin, CRP	D-dimer, CRP, IL-6, IL-8, Ferritin	D-dimer, CRP, IL-6, IL-8, Ferritin	Lymphopenia, thrombocytopenia, CRP, D-dimer, IL-6	Lymphopenia, thrombocytopenia, CRP, D-dimer, IL-6	Lymphopenia, CRP, D-dimer	9/9
Other microbial cause	No	No	No	No	No	No	No	No	No	No	0/10
Evidence of COVID-19	No	Yes	Yes	Yes	Yes	Yes	Yes	Yes	No	Yes	8/10
Other clinical features	Meningeal signs	–	Myalgia	Ileitis, ascites, pleural effusions	Acute hypoxemic respiratory failure, cardiogenic shock with biventricular, dysfunction, ileitis	–	–	–	Headache	Toxic shock syndrome, cytokine storm	6/10
Treatment	IVIG, mPDN	Milrinone, IVIG, methylprednisolone, aspirin, ceftriaxone, NIV	Dopamine, noradrenaline, milrinone, IVIG, methylprednisolone, aspirin, ceftriaxone, clindamycin, NIV	Noradrenaline, adrenaline, milrinone, IVIG, methylprednisolone, heparin, ceftriaxone, clindamycin, metronidazole, MV	Amoxicillin-clavulanic, methylprednisolone, immunoglobulin, cytokine hemadsorption, vasopressor, inotropic support	Methylprednisolone, anakinra, lopinavir/ritonavir, broad spectrum antibiotics, LMWH	Methylprednisolone, anakinra, lopinavir/ritonavir, broad spectrum antibiotics, LMWH	Methylprednisolone, IVIG, noradrenaline, anakinra	Methylprednisolone, IVIG, anakinra, LMWH	Milrinone, norepinephrine, empiric antibiotic, enoxaparin, Vitamin K, tocilizumab, convalescent plasma, remdesivir, IVIG, steroids	–
Admission duration (days)	11	8	8	8	20	14	10	8	10	–	Average 11 days
Response	Yes	Yes	Yes	Yes	Yes	Yes	Yes	Yes	Yes	Yes	10/10

† Table note missing.

CRP: C-reactive protein; IVIG: Human intravenous immunoglobulin; LMWH: Low molecular weight heparin; MV: Mechanical ventilation; NIV: Non-invasive ventilation; NP: Not provided; RRT: Renal replacement therapy; VA-ECMO: Veno-arterial extracorporeal membrane oxygenation.

### Performance-based treatment considerations

#### Gastrointestinal symptoms

Considering that gastrointestinal symptoms are the most common clinical manifestations of MIS-C (87% of patients) [43], supportive care such as a proton pump inhibitor should first be given to provide gastric protection in children exhibiting gastrointestinal symptoms. It is worth noting that Zonulin is a regulator of tight intercellular epithelial junctions [44]. In MIS-C, local mucosal inflammation of the gastrointestinal tract caused by SARS-CoV-2 leads to increased zonulin release and subsequent increased intestinal permeability thereby amplifying the permissive effect on SARS-CoV-2 antigen, which in turn leads to inflammatory immune activation [45]. larazotide, a zonulin antagonist, was shown by Lael *et al.* to attenuate the cytokine storm and reduced the degree of SARS-CoV-2 antigenemia, thereby improving clinical symptoms in individual patients [4,45,46]. In addition, Khesrani *et al. *suggested that children with atypical abdominal pain syndrome should undergo an abdominal CT angio-scan for evaluation of the condition to establish an appropriate medical treatment (immunoglobulins, glucocorticoids) to prevent digestive complications [47,48].

#### Cardiac dysfunction

During the acute inflammatory phase of the disease, children may experience a series of cardiac dysfunction such as electrical abnormalities, myocarditis, valve dysfunction, coronary aneurysms or dilation, heart failure and shock [49]. Monitoring of brain natriuretic peptide and troponin levels, electrocardiogram and serial echocardiographic assessment of cardiac function can help guide therapy. Management focuses on supportive care to prompt to minimize the risk for cardiac decompensation. We also recommended that all patients with cardiac involvement receive IVIG and glucocorticoids.

At present, recommendations for follow-up of cardiology in patients with MIS-C are still evolving. Patients with conduction abnormalities on examination should be seen in telemetry during their hospitalization [36]. Continuous telemetry monitoring can quickly detect and treat patients with heart abnormalities to a certain extent. Positive inotropic drugs and intravenous diuretics such as dobutamine, dobutamine and milrinone are recommended for patients with severe cardiac insufficiency. In cases of the fulminant disease, it may be necessary to obtain mechanical hemodynamic support such as veno-arterial extracorporeal membrane oxygenation or a ventricular assist device in the pediatric intensive care unit (PICU) [17,50].

#### Shock

When the patient is in shock, resuscitation should be in settings with access to the intensive care unit. Resuscitation methods include giving inotropic support, using mechanical ventilation for respiratory support and so on. For patients who require multiple inotropic and/or vasopressors, high-dose intravenous glucocorticoids (10–30 mg/kg/day) are the best method. When children with MIS have fluid-refractory shock, the first choice is vasoactive agents such as epinephrine or norepinephrine. When there is evidence of left ventricular dysfunction, epinephrine is preferred [38,51].

#### Acute kidney injury

Although pediatric cases often have a mild course, some patients with MIS-C will still have acute kidney injury or even acute renal failure in the clinic. Currently, cytokine storm syndrome has been postulated as one cause of multiple organ dysfunction, including renal function impairment. For the above reasons, Ronco *et al.* proposed that the use of hemofiltration or hemoperfusion containing HA330/HA380 cartridges may provide support for various organs [52]. Additionally, in the absence of resources for hemoperfusion, continuous kidney replacement therapies are recommended as a supportive treatment, but it is not available as an alternative therapy [53].

### Immune-modifying therapies

#### IVIG

The exploration of the mechanisms of IVIG in MIS-C is focused on the following aspects. Cytologically, IVIG affects mainly neutrophils in blood leukocytes, reducing their number and their profile by more than 50% [23] and IL-1b neutrophils by more than 90% [54]. IVIG-induced neutrophil death is not associated with apoptosis, as this process is not blocked by pancysteine inhibitors [55]. The reduction in IL-1β+ neutrophils was predominant after treatment, whereas the total number of NEP and IL-1β-negative mature neutrophils remained essentially unchanged; therefore, mature IL-1β+ neutrophils could be a target for the therapeutic regimen of IVIG. In terms of cellular immunology, IVIG had pleiotropic effects on cell surface marker expression and neutrophil maturation according to flow cytometry data, a finding that was associated with an increase in granulocyte cell surface CD10 and CD49d and a decrease in CD15 and CD101 during IVIG treatment. Furthermore, the non-replication of the Fc fragment would inhibit the effect of IVIG on human neutrophils, which would play an important role in mediating the clinical effects of IVIG on the neutrophil spectrum [23]. In addition, IVIG induces autophagy in peripheral blood mononuclear cells through F(ab’)2 and PI3K-dependent pathways, so future studies should be aimed at the PI3K pathway [54]. Given that immunoglobulins confer a variety of immune effects on the human body, including anti-inflammatory effects, passive immunity and immunomodulatory. Therefore, IVIG is recommended for all patients who meet the diagnostic criteria of MIS-C. It is administered as either 2 g/kg once within 8 to 12 h or 1 g/kg twice within 24 h, according to the assessment of the patient's physical condition [49]. It is worth mentioning that the current evidence for the use of IVIG to treat MIS-C patients is limited to case series. In clinical cases, the overwhelming majority of patients receiving IVIG treatment have improved and restored normal physiological functions [37,56].

#### GC

GC is the most important regulatory hormone of the body's stress response, and it is also the most widely used and effective anti-inflammatory and immunosuppressive agent in clinical practice. Because the symptoms of MIS-C patients are highly similar to cytokine storm syndrome, GC is proposed as a treatment method for MIS-C. Currently, IVIG alone is the main treatment for patients with mild MIS-C. The combination of glucocorticoids and IVIG is commonly used in patients with more severe MIS-C clinical symptoms. Glucocorticoids are often not used alone in patients with mild MIS-C due to their significant side effects, but only as a substitute for IVIG when it is not available [57]. A retrospective and cohort study showed that the recovery time of heart function in MIS-C patients receiving immunoglobulin combined with glucocorticoid therapy is faster than that of MIS-C patients receiving immunoglobulin therapy alone [58]. For patients with shock and/or organ-threatening disease, low-to-moderate–dose GCs such as intravenous methylprednisolone (1–2 mg/kg/day) should be used as adjunctive therapy with IVIG. Furthermore, for patients who do not respond to IVIG and low-to-moderate–dose GCs, consider high-dose intravenous methylprednisolone (10–30 mg/kg/day, the maximum dose is 1 g) [36]. As expected, in most cases, the clinical status gradually improved until the fever subsided after receiving different doses of GCs [40,50,59]. Patients can be switched to an equivalent dose of oral GCs such as prednisolone or prednisone at discharge once they have recovered their physical function and then tapered over 2 to 4 weeks [51].

#### TNF inhibitors

TNF-α is a pro-inflammatory cytokine, and it has been shown that TNF-α is abnormally elevated in patients with MIS-C and plays an important role in the pathogenesis of MIS-C in the medium term [60,61]. Therefore, TNF-α is an optional therapeutic target. Infliximab, a TNF-α blocker, is now used as second-line therapy for MIS-C patients with persistent inflammation or myocardial dysfunction, often in PICU patients [62,63]. The vast majority of PICU patients who received high-dose infliximab (10 mg/kg) in combination with IVIG controlled the inflammatory process and had improved cardiac function [64]. Therefore, drug combination regimens may become a trend in future MIS-C treatment, but there is a need to determine whether a combination drug approach can maximize treatment efficacy while minimizing adverse effects [31].

#### Anticoagulant therapy

Due to the hypercoagulable state associated with COVID-19, patients with MIS-C are at risk for thrombotic complications, including pulmonary embolism and deep vein thrombosis [65]. For most MIS-C patients, anticoagulant therapy should tailor to the patient's thrombosis risk. To prevent thrombosis, low-dose aspirin (3–5 mg/kg/day) is recommended for all patients with MIS-C, excluding those with active bleeding, significant bleeding risk, and/or platelet counts ≤80,000/μl [36]. In addition, MIS-C patients with a coronary z-score greater than 10.0 should be treated with therapeutic anticoagulants such as enoxaparin (factor Xa level: 0.5–1.0), and warfarin. Patients with severe left ventricular dysfunction are at greater risk of intracardiac thrombosis, and therapeutic anticoagulants are recommended [66,67].

### Adjuvant therapy

#### Hemadsorption

The clinical manifestations and laboratory features of excessive inflammation in children are similar to adult patients with COVID-19, supporting the hypothesis that MIS-C is caused by the immune-mediated damage of SARS-CoV-2 [39]. Hypercytokinemia is one of the causes of excessive inflammation. The extracorporeal cytokine hemadsorption device has been used for cytokine storm-related excessive inflammation because it can achieve the safe and rapid reduction of cytokine levels. Therefore, it is approved for use in critically ill patients with COVID-19. In the clinic, after using blood adsorption, rapid improvement in shock and multiple dysfunction parameters can be observed without related side effects [68].

#### Anti-IL-1 antagonists

In order to reduce excessive inflammation in patients with MIS-C, in addition to using widely non-specific Antagonists, targeted therapies that precisely target inflammatory cytokines are often used. Hadjadj *et al.* showed that in adult patients with severe COVID-19 disease, IL-1 plays an important role in the pathogenesis of excessive inflammation. Therefore, IL-1 receptor antagonists have become one of the first-choice therapeutic agents to control and reduce inflammation in patients with MIS-C [69]. Anakinra is an IL-1 receptor antagonist with a strong anti-inflammatory effect and can down-regulate the downstream proinflammatory cascade secondary to IL-1. Even in the case of acute infection, the safety of anakinra is very good. As an extrapolation of IVIG-resistant KD, anakinra has been used by some doctors in MIS-C, and has achieved good therapeutic effects in clinical cases [40,41].

#### Anti-IL-6 antagonists

IL-6 is a major pro-inflammatory cell. Because its level is significantly elevated in many MIS-C patients, it is used as an optional therapeutic target [70]. Tocilizumab is a recombinant IL-6 receptor antagonist, which can inhibit IL-6 activity and may prevent the destruction of immune tolerance. It is not yet possible to draw any conclusions about the function of tocilizumab in MIS-C patients. However, among adult patients with severe COVID-19, IL-6 inhibitor drugs have been shown to have a certain effect on reducing the mortality of severely ill patients [71,72]. However, at present, there is no clear dosage and administration method for the clinical use of IL-6 inhibitor drugs. Clinicians should consider using IL-6 inhibitor drugs in perfect clinical trials to evaluate their risks and benefits [42]. Likewise, other future drug applications for MIS-C patients are still being tested and explored, therefore, we summarize 5 studies on future drug clinical trial data for patients with MIS-C (Table 3).

**Table 3.** Clinical application and outcome of future trial drugs of MIS-C.

Study	Group 1 [59]	Group 2 [61]	Group 3 [50]	Group 4 [48]	Group 5 [64]
Patients (n)	58	16	20	44	186
Male	38 (66)	8 (50)	10 (50)	20 (45)	115 (62)
Age (median in years, [IQR])	9	10	10	7	8
Treatment					
Intravenous immunoglobulin	41 (71)	15 (93)	20 (100)	36 (82)	144 (77)
Glucocorticoids	37 (64)	4 (25)	2 (10)	42 (96)	91 (49)
TNF inhibitors	8 (14)	0 (0)	0 (0)	0 (0)	0 (0)
Anti-IL-1 antagonists	3 (5)	1 (6)	1 (5)	8 (18)	24 (13)
Anti-IL-6 antagonists	0 (0)	1 (6)	1 (5)	0 (0)	14 (8)
Outcomes					
Recovery	57 (98)	16 (100)	20 (100)	43 (98)	134 (72)
Death	1 (2)	0 (0)	0 (0)	0 (0)	4 (2)

### Novel coronavirus vaccine & MIS-C

Because the pathogenesis of MIS-C is related to infection with the SARS-CoV-2, an effective SARS-CoV-2 vaccine may lead to the occurrence of MIS-C. If the vaccine can induce this kind of antibody response in children, healthy children will face a potentially huge risk to get a vaccine designed to prevent SARS-CoV-2 disease [73]. According to reports, the possibility of MIS-C after SARS-CoV-2 infection is roughly 1 in 4000 [74]. At present, there are over 10 million children vaccinated in the United States. But currently, only Dr. Nygaard has reported a patient who was infected with SARS-CoV-2 after receiving the mRNA vaccine [75]. Although the cause of MIS-C is still uncertain, it is still an extremely rare condition even if it is indeed caused by mRNA vaccination. Therefore, it is recommended to pay special attention to it for timely treatment and further research.

## Conclusion

MIS-C may be a delayed immune phenomenon caused by SARS-CoV-2 infection. This article focuses on treatments for the clinical manifestations of patients with severe complications, discusses the advantages of different treatment options, and presents a statistical analysis of the clinical efficacy of various treatment options. Because of the special status of the heart in the human body, cardiac dysfunction is often an urgent problem, and the damage to the heart by MIS-C in clinical practice is changeable and complex, which requires doctors to have keen clinical judgment ability.

## Future perspective

For emerging adjuvant treatments, such as hemadsorption, and IL inhibitors, clinical samples have not yet clearly demonstrated their effectiveness. Reasonable suspicion, timely diagnosis, and precise treatment will maximally bias the patient's outcome in a better direction.

**Executive summary**

**Pathogenesis of MIS-C**

- Although the MIS-C usually appears 2–4 weeks after infection with SARS-CoV-2, the pathogenesis of MIS-C is not clear, it may be a delayed immune phenomenon associated with inflammation.

**Basic treatment of MIS-C**

- Most patients with moderate and severe phenotypes are treated first with intravenous immunoglobulin (IVIG) and glucocorticoid (GC) therapy.

**Performance-based treatment considerations**

- Considering that gastrointestinal symptoms are the most common clinical manifestations of MIS-C (87% of patients), supportive care such as a proton pump inhibitor should first be given to provide gastric protection in children exhibiting gastrointestinal symptoms.

**Immune-modifying therapies**

- Given that the symptoms of MIS-C are similar to those of KD, most of the initial treatment options chose IVIG as the first-line treatment for patients, and the case series achieved good results.

**Adjuvant therapy**

- The extracorporeal cytokine hemoadsorption device has been used for cytokine storm-related excessive inflammation because it can achieve the safe and rapid reduction of cytokine levels.
